# Longitudinal comparison of ePRO satisfaction ratings in patients with cancer receiving systemic or radiotherapy

**DOI:** 10.1186/s41687-026-01000-9

**Published:** 2026-01-31

**Authors:** Ian Kudel, Annette Christianson, Jordan Jackson, Zoya Shamsi, Sushil Beriwal

**Affiliations:** Varian, A Siemens Healthineers Company, Palo Alto, USA

**Keywords:** Patient reported, Electronic, Symptoms, Satisfaction, ePRO

## Abstract

**Background:**

ePROs implementation for patients in active treatment for cancer requires that the app be nonburdensome and that it does not incumber clinical care team (CCT) workflows. Therefore, comparing longitudinal patient satisfaction of a single solution for those receiving radiation therapy (RT) and systemic therapies (ST; chemo-, immuno-, and hormonal) is critical to ensuring that maximal clinical benefit can be achieved with minimal impact to the provision of care.

**Methodology:**

Active treatment ePRO users with working active accounts > 30 days, who answered the question “How likely are you to recommend (software name) to another patient” using an 11-point scale twice or more (*n* = 7,202), were included. At a minimum all participants had access to three features: 1) symptom-reporting (administered at specific intervals or ad-hoc) via CTCAE-derived questionnaires or health-related quality of life questionnaires, 2) a diary to record any personal information, and 3) a secure communication feature for corresponding with the CCT. Additional data were passively collected by the app. All variables were analyzed descriptively and via a negative binomial mixed model (NB) with patient as a random effect and repeated measurements with an autoregressive covariance structure.

**Results:**

Patients (mean age=61.7 years, SD=11.8) were from the US (n=4,941; sites=12), Canada (n=1,700; sites=4) and Europe (n=561; sites=4). Additionally, most were English speakers (n=6,794; 94.3%), used a smartphone (n=5,480; 76.1%), and received systemic therapy (n=6,468; 89.8%). The mean number of app satisfaction reports per patient was 3.1 (SD=1.6; range 2-13). The mean rating at Time 1 was 8.5 (SD=2.5), which is high, and it increased slightly over time. The NB mixed model found that satisfaction levels for patients receiving radiation did not significantly differ (p=0.835) from those receiving systemic therapy after adjusting for the other variables indicating that, over time, patients regardless of the treatment reported similar app satisfaction scores.

**Conclusion:**

These findings suggest that a single ePRO solution can be deployed with similarly high levels of patient satisfaction across two widely used treatment modalities, despite health system, practice, and ePRO deployment differences, minimizing CCT workflow and patient burden.

## Introduction

The increasing ubiquity of electronics (smartphones, computers, and tablets), patient-friendly software, and connectivity (e.g., cellular, Wi-Fi) has changed the collection of patient-reported data in clinical oncology settings from pen-and-paper surveys collected at the point of care to cloud-based, bidirectional digital apps (ePRO) that facilitate patient-clinical care team (CCT) communication of cancer treatment symptoms/severity and clinical recommendations. To further improve patient and CCT usability, these apps are not limited to this one purpose, but can include portal functionality (e.g., appointment scheduling, reviewing test results) and electronic health record integration. These complex tools require regular assessment of patient experiences to ensure that the software meets stakeholder needs but, to date, there has been limited study quantifying this information [12]. Fortunately, this can be easily addressed and has the potential to introduce a new frontier in the collection and use of patient-reported data.

There is increasing evidence that frequent and effective patient-provider communication of treatment-related symptoms via electronic patient-reported outcome solutions (ePROs) optimizes symptom management [15], which can improve treatment adherence and impact downstream outcomes including time on treatment, survival, and quality of life, while at the same time decreasing costs [1, 4, 17]. Generally, it is believed that greater usage is ideal, but real-world challenges can impede greater engagement and generally fall into three categories: (1) patient factors (e.g., sociodemographics such as age and health; [3]), (2) clinical-care team factors (e.g., workflows and lack of timely or relevant clinical care team responses; [18]), and (3) software (e.g., the usability and the interface; [2, 15]). Ideally, an app should meet patients’ needs across the cancer-care continuum, but perhaps most importantly during active treatment, when they are most likely to be experiencing treatment-related effects associated with radiotherapy (RT) and/or systemic therapy (ST; chemo-, immuno-, and hormonal). Interestingly, to date, there are only a handful of papers regarding ePRO use across treatment methodologies [16, 19, 20] and none testing whether patients find a single solution acceptable despite differences in treatment administration and their impact. Generally, ST is administered at regular intervals (e.g., monthly) and has whole body side effects (e.g., nausea, vomiting, fatigue) which usually peak at specific timepoints (e.g., a week after an infusion). Conversely, RT is administered 4 to 5 days/week for a set period of time (e.g., 6 weeks), and patients usually experience distress at the site of treatment, with symptoms progressively worsening over time, peaking near or at the conclusion of active treatment.

Usability testing is often used to evaluate solution acceptability before its release; however, because of the qualitative, experiential interviewing required to assess acceptability, the number of patients included in these evaluations tends to be smaller. A complement to this approach is broadly and directly eliciting patient perspectives through the regular collection of patient satisfaction ratings, a behavioral manifestation of the usage experience. The medical establishment has embraced this approach, and patients with cancer are periodically asked to complete questionnaires for an array of services including, but not limited to, general cancer care [11, 22], telemedicine appointments [9], and wound care [8, 10, 14]. Therefore, extending this methodology to include the ePRO experience is reasonable. In this case high satisfaction is most likely to be a reflection of app utility; patients are more likely to give high ratings when they find the software meets their needs and poor ratings when there is little or no obvious benefit [5, 7]. To date, empirical evidence exploring the factors associated with ePRO app satisfaction scores is limited, but there are indications that regardless of how the app is employed by the CCT, it is not impacted by the age of respondent [12], the geographical region in which the patient is receiving treatment, or the body location of the radiation treatment, [13]. This study extends the literature by testing whether patients receiving active RT or ST systematically report different levels of satisfaction during this critical period when they are experiencing deleterious treatment-related effects and thus are more likely to be reliant on the app to communicate their distress with the hopes of receiving CCT guidance to remediate at least some of it.

## Methods

### Participants

Active ePRO users, defined as those who have had an active account for more than 30 days and responded to the app satisfaction item at least twice (*n* = 7,202), were included in this study.

### ePRO platform

The ePRO is a multifunction, cloud-based solution that has been installed in 204 oncology clinics across 10 countries, is available in 12 languages, and has over 183,000 active users. Clinical staff at each site onboard patients by assisting them with creating a patient profile and training them regarding its use. The vast majority of sites utilize the ePROs base configuration, which includes three components; the treatment symptom reporting tool, secure messaging, and the diary [6]. The treatment symptom reporting tool is the most critical component; most providers choose to administer treatment-specific questionnaires, called modules, at prespecified points when patients are most likely to be exhibiting symptoms, but patients are able to respond to them ad hoc when symptoms arise at any point during treatment. These modules are structured in the following manner. At the beginning of each, respondents are asked to report their overall status using an 11-point visual analog scale (0 = None to 10 = Worst Possible), changes in their weight, and report their physical functioning using an Eastern Cooperative Oncology Group Performance Status Scale-based item and a 5-point Likert scale. Next, patients are given the option of selecting the general symptom domain in which they are experiencing concerns and are then presented with non-technical items that incorporate the three-response-level Common Terminology Criteria for Adverse Events (CTCAE, version 5; 2017). CTCAE Grades 1 through 3 are replaced with the more patient-friendly Mild, Moderate, and Severe. Generally, when a patient indicates a moderate or severe symptom, a real-time color-coded electronic alert prompts the CCT to offer clinical recommendations via the app. Separate medical oncology modules have been developed for monotherapy (chemo-, immuno- or hormone therapy) and combination therapy while radiotherapy modules focus on specific treatment site locations (e.g., breast, prostate, bone). All modules, regardless of the treatment focus, also include a free text option so that patients can report less frequent symptoms.

Patients also have access to a secure messaging feature, which functions as an internal email system connecting patients and the CCT. When it is accessed, the patient is asked to select whether the message is clinically- or non-clinically focused and then they are provided with a free-text box to enter their message. The CCT then responds to those emails. In the third feature, patients have access to a Diary to record private treatment-related information. The CCT can only access the stored information stored when there is a patient-safety concern, which is defined as a “Severe” (Grade 3) symptom.

Additional features, which give patients access to other tools include the ability to view laboratory test results, appointments (seeing them and adjusting them), prescribed medication, and clinical documents relevant to their cancer treatment. Finally, the educational feature gives CCTs the ability to send any information, including treatment-related information to patients in the form of PDFs, videos, or web links.

### Ethical considerations

The WCG IRB Affairs Department (www.wcgclinical.com) determined on July 25, 2024 that this project was exempt under 45 CFR § 46.104(d)(4) because the data were archival and did not include protected health information.

### Variables

#### Patient satisfaction

Active ePRO users are randomly selected to answer the question, “How likely are you to recommend (ePRO name) to another patient?” using an 11-point visual analog scale ranging from “Unlikely” (a zero) to “Very likely” (a ten). Patients who respond with a numerical rating are also given the option to include comments in a free-text box. Those who choose not to respond can opt out by clicking on a large, visible “X” in the upper right side of the question box.

#### Age at ePRO enrollment

The ePRO passively collects the patients’ birth year. Approximate age was calculated by subtracting the year (2024) from the birth year.

#### Treatment modality

All patients are assigned a module that includes symptoms that are likely to occur when receiving their treatment regimen. This study included patients receiving either systemic therapy or radiotherapy. This information is saved on the clinic-facing side of the app and also passively collected on the patient-facing side. For this study treatment modality was extracted from the patient-facing component and the variable was extrapolated based on this information.

#### Device

Patients can access the ePRO using either a smartphone or a computer. The ePRO experience is the same across all devices, but since smartphones have a much smaller screen than tablets or PCs, it may impact overall usage experience. Therefore, the passively collected information (device and operating system) has been dichotomized into two groups, smartphone and computer.

#### Language

The ePRO can display 12 languages (list), but the most widely used is English, so this variable is dichotomized into English and other language.

#### Geographic region

Users are based in one of three regions (the United States, Canada, and Europe); thus this variable is trichotomized.

### Analyses

The analyses are divided into two parts. In the first, all study variables are reported descriptively. Categorical variables are reported using counts and percentages and continuous variables are reported using mean, standard deviation, and range. To facilitate understanding of patient ratings over time a simplified alluvial flow diagram presents trichotomized satisfaction scores based on widely used Net Promoter Score classifications: the groups are low (scores of 0–6), medium (scores of 7 and 8), and high (scores of 9–10). The diagram includes only the first five timepoints due to the decreasing sample size, which makes it difficult to see the changes beyond that timepoint.

In the second, a generalized linear mixed model, specifically a negative binomial model (NB) with a log link function, was used to test the association between patients’ satisfaction ratings and treatment modality (radiotherapy or systemic), with user as a repeated measure. This model best represents multiple measurements per person of an outcome with a skewed distribution and only discrete positive values. The model also controlled for age (continuous), operating system (smartphone or computer), language (English or other), geographical area (US, Canada, or Europe), and the timepoint of the reported rating (continuous). Based on log likelihood testing, a first-order autoregressive covariance structure was chosen to model the longitudinal nature of the data because it demonstrated a better fit compared to other options (compound symmetry, first-order antedependence, or unstructured structures). The standard regression parameters and odds ratios (ORs) with 95% confidence intervals (95% CIs) are reported. Additionally, estimated marginal means (EMM; for categorical variables) and estimated marginal predictions (EMP; for continuous variables) were calculated to show mean ratings for satisfaction when adjusting for all other variables in the model, using unbalanced populations and exponentiating in order to report in the parameters in the original metric. EMM were reported for each level of the variables. EMP were reported at the mean and quartile values for age and are reported overall and by treatment type for the first five timepoints because they include at least 10% of baseline respondents.

All statistical analyses were performed using SAS version 9.4 and R, with 2-sided hypothesis testing and a p-value of less than 0.05 as the criterion for statistical significance.

## Results

### Descriptive statistics

The majority of ePRO users were older (mean = 61.7 years), English-speaking (94.3%), located in the US (68.6%), undergoing systemic treatment (90.9%), and used the ePRO on their phones (76.1%; Table 1). The mean number of ratings per person was 3.1 (SD = 1.6; range 2–13) and the mean satisfaction scores incrementally increased at each timepoint (Table 2).

**Table 1:** Patient-level summary statistics for patients with at least 2 satisfaction scores

Variable	*n*	%
Categorical		
Device		
Smartphone	5,480	76.1
Computer	1,722	23.9
Language		
English	6,794	94.3
Other	408	5.7
Treatment		
Radiation	734	10.2
Systemic	6,468	89.8
Geographic Region		
United States	4,941	68.6
Canada	1,700	23.6
Europe	561	7.8
	**Mean (SD)**	**Range**
Continuous		
Age	61.7 (12.0)	18–90
Number of Satisfaction Responses	3.1 (1.6)	2–13

**Table 2:** Summary statistics and distributions of satisfaction scores at each timepoint

**Timepoint**	**1st**	**2nd**	**3rd**	**4th**	**5th**	**6th**	**7th**
n	7,202	7,202	3,725	1,968	1,053	576	330
Mean	8.50	8.58	8.72	8.84	8.93	8.93	9.01
SD	2.46	2.45	2.37	2.29	2.10	2.10	2.04
0	219 (3.0)	228 (3.2)	111 (3.0)	57 (2.9)	17 (1.6)	10 (1.7)	5 (1.5)
1	54 (0.8)	66 (0.9)	31 (0.8)	10 (0.5)	4 (0.4)	5 (0.9)	3 (0.9)
2	82 (1.1)	73 (1.0)	30 (0.8)	21 (1.1)	13 (1.2)	6 (1.0)	3 (0.9)
3	86 (1.2)	67 (0.9)	32 (0.9)	16 (0.8)	13 (1.2)	4 (0.7)	3 (0.9)
4	90 (1.3)	84 (1.2)	36 (1.0)	12 (0.6)	8 (0.8)	2 (0.4)	1 (0.3)
5	417 (5.8)	361 (5.0)	161 (4.3)	67 (3.4)	36 (3.4)	22 (3.8)	9 (2.7)
6	162 (2.3)	174 (2.4)	87 (2.3)	28 (1.4)	16 (1.5)	3 (0.5)	5 (1.5)
7	388 (5.4)	349 (4.9)	158 (4.2)	96 (4.9)	50 (4.8)	29 (5.0)	14 (4.2)
8	838 (11.6)	752 (10.4)	348 (9.3)	177 (9.0)	92 (8.7)	57 (9.9)	27 (8.2)
9	734 (10.2)	757 (10.5)	376 (10.1)	181 (9.2)	95 (9.0)	63 (10.9)	39 (11.8)
10	4,132 (57.4)	4,291 (59.6)	2,355 (63.2)	1,303 (66.2)	7,09 (67.3)	375 (65.1)	221 (67.0)
**Timepoint**	**8th**	**9th**	**10th**	**11th**	**12th**	**13th**	
n	183	108	45	13	6	1	
Mean	9.11	9.13	9.29	9.31	9.50	10.00	
SD	1.76	1.96	2.06	2.21	0.84		
0	2 (1.1)	2 (1.9)	1 (2.2)	0 (0)	0 (0)	0 (0)	
1	0 (0)	0 (0)	0 (0)	0 (0)	0 (0)	0 (0)	
2	0 (0)	0 (0)	1 (2.2)	1 (7.7)	0 (0)	0 (0)	
3	0 (0)	0 (0)	0 (0)	0 (0)	0 (0)	0 (0)	
4	2 (1.1)	3 (2.8)	1 (2.2)	0 (0)	0 (0)	0 (0)	
5	6 (3.3)	5 (4.6)	0 (0)	0 (0)	0 (0)	0 (0)	
6	7 (3.8)	0 (0)	0 (0)	0 (0)	0 (0)	0 (0)	
7	13 (7.1)	3 (2.8)	0 (0)	0 (0)	0 (0)	0 (0)	
8	11 (6.0)	6 (5.6)	1 (2.2)	0 (0)	1 (16.7)	0 (0)	
9	11 (6.0)	10 (9.3)	6 (13.3)	1 (7.7)	1 (16.7)	0 (0)	
10	131 (71.6)	79 (73.2)	35 (77.8)	11 (84.6)	4 (66.7)	1 (100.0)	

**Fig. 1:**
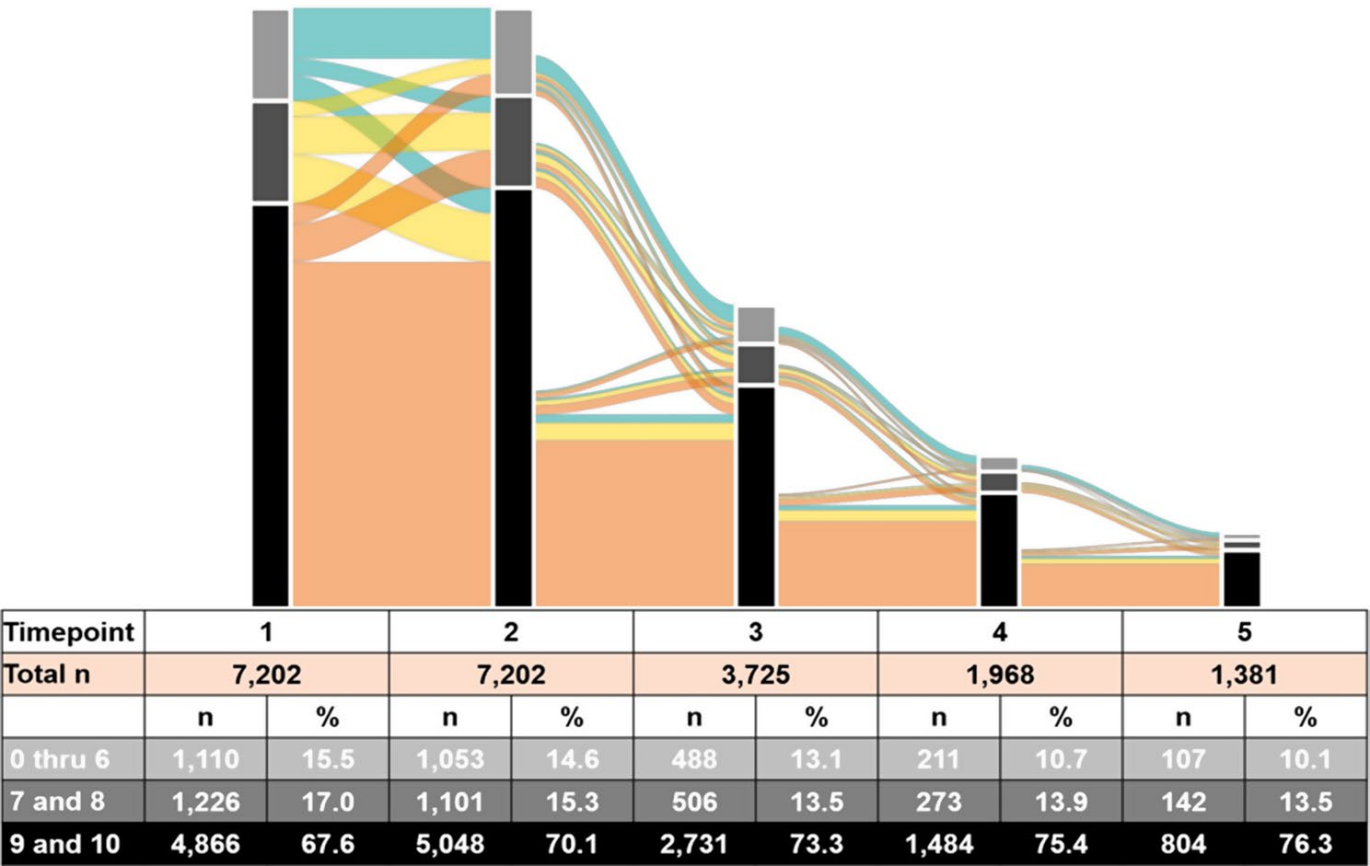
Alluvial chart showing NPS satisfaction scores for the first five survey timepoints, with longitudinal movement in subsequent responses. The columns show the distribution of ratings at each timepoint, with height based on the number of participants and color of each section matching the category in the associated table. The colored sections between columns indicate the transition from one rating to the subsequent one, while the colors represent patient response at the first timepoint; thickness of the section reflects the number of respondents in that path. For example, more than half of respondents (n=4,156,57.5%) rated the app a 9 or 10 (highly satisfied) for their first two reports. Therefore, the light orange section connecting them is appropriately sized to reflect that percentage of the total. As the overall number of participants reporting satisfaction decreases, the column height decreases in size proportionally

The alluvial diagram (Fig. 1) demonstrates that a high and slightly increasing proportion, more than two-thirds at each timepoint, provided high ePRO ratings (9 or 10), while a low and slightly decreasing proportion, at most 15.5%, rated the ePRO low (0 to 6). Most of those (85.4%) who rated it high in their first measurement continued to do so in their second, while there was also a fair amount of movement to high from those who originally rated it medium (46.5%) or low (26.8%). After the second measurement, the number of respondents began to fall off sharply with each successive measurement.

### NB model

The primary variable of interest, treatment type, did not significantly differ with regard to whether patient received systemic or radiation treatment (OR 1.00, 95% CI 0.95–1.02). A comparison of the other variables found that patient ratings were significantly lower in Canada as compared to the US (OR 0.93, 95% CI 0.90–0.96), and higher for users accessing the ePRO on phone as compared to computer (OR 1.04, 95% CI 1.02–1.06). Scores showed a significant but small increase over subsequent measurements (OR 1.00, 95% CI 1.00–1.00), and a significant but small decrease as years of age increased (OR 1.00, 95% CI 1.00–1.00). None of the other comparisons were significant (Table 3). All the adjusted means and predictions were between 8 and 9 (Tables 4 and 5), indicating a high level of satisfaction across all levels of the included factors, which further supports the finding that they are consistently high and that the statistically significant variables are not meaningfully different. For example, the EMP at the mean age was 8.51 (95% CI 8.71–9.13). The value for 18-year old patients was 8.91 (95% CI 8.46–8.56) and for 90 year-olds it was 8.26 (95% CI 8.71–9.13), which is a small decrease (0.65 points) across a 72-year range.

**Table 3:** Coefficients and confidence intervals (95%) for predictors in NB mixed model predicting satisfaction scores

			Confidence Intervals (95%)		
Factor	Comparison	B	Lower Limit	Upper Limit	*p*-value
Timepoint of rating		0.05	0.04	0.07	< 0.01
Age at ePRO enrollment		-0.01	-0.01	-0.01	< 0.01
Smartphone	vs. Computer	0.27	0.15	0.39	< 0.01
English Language	vs. Other Language	0.28	-0.10	0.66	0.14
Radiotherapy Treatment Modality	vs. Systemic	0.02	-0.17	0.22	0.81
Geographical Region	vs. USA				< 0.01
Canada		-0.38	-0.05	-0.26	
Europe		0.09	-0.27	0.44	

**Table 4:** Adjusted EMMs and EMPs of satisfaction scores for variables included in the NB mixed model

Covariate	Value	Prediction	95% CI
Continuous			
Age (years)	Minimum (18)	8.91	8.71–9.13
	Mean (61.7)	8.51	8.46–8.56
	Maximum (90)	8.26	8.12–8.39
Categorical			
Operating system	Phone	8.57	8.51–8.63
	Computer	8.30	8.19–8.41
Language	English	8.52	8.47–8.58
	Other	8.25	7.90–8.63
Treatment type module	Radiation	8.55	8.35–8.75
	Systemic	8.50	8.45–8.56
Geographic Region	USA	8.58	8.52–8.65
	Canada	8.20	8.09–8.31
	Europe	8.66	8.32–9.02

**Table 5:** Adjusted emps of satisfaction scores at first five timepoints overall and by treatment type

Treatment	Measurement number	Prediction	95% CI
Overall	1	8.42	8.35–8.48
	2	8.48	8.43–8.53
	3	8.54	8.49–8.60
	4	8.61	8.54–8.68
	5	8.67	8.58–8.77
Radiation	1	8.45	8.26–8.65
	2	8.52	8.32–8.72
	3	8.58	8.38–8.79
	4	8.65	8.44–8.86
	5	8.71	8.50–8.94
Systemic	1	8.41	8.35–8.48
	2	8.48	8.42–8.53
	3	8.54	8.48–8.60
	4	8.60	8.53–8.68
	5	8.67	8.58–8.76

## Discussion

The dynamic nature of ePROs represents a paradigmatic shift in the collection of patient-reported symptom data, but any successful long-term solution requires that the software minimally burdens CCT workflows and sick patients. Therefore, it is incumbent on developers to assess its utility, which was not a requirement when such information was collected via paper-and-pencil administration of questionnaires. In this case, we used a sophisticated analytic plan to evaluate a global, longitudinal ePRO satisfaction dataset collected from a very large, international sample of active treatment patients and found stable, high scores and no meaningful difference in app satisfaction across two disparate treatment modalities, both of which patients receive (sequentially or at the same time) as part of the standard of care treatment.

While null findings do not prove equivalence, sample size and similarities in the adjusted satisfaction scores (EMMs and EMPs) indicate a lack of a practical difference. The variation of site ePRO deployments, health system-, practice-, and patient-level differences combined with the literature regarding app usage in patients with cancer [5, 7] lead us to hypothesize that the primary driver for the consistently high ratings was likely app utility; the ePRO met patients’ needs.

We acknowledge that to achieve our study goals we made a number of methodological concessions. Notably, we could not include any sociodemographic (other than age), clinical, or treatment data because the ePRO app does not capture any protected health information; those data reside in the EHR. This is intentional, and for security reasons will not change. While it could be possible to coordinate EHR and satisfaction data across all sites the differing data privacy requirements make it impractical from cost and timeline perspectives. Further, the generic wording of the satisfaction item makes it difficult to determine what factors are influencing the ratings. While more specific wording could provide additional insight, the consistently high scores, regardless of how respondents interpret the item, suggest that patients are getting what they need from the software. Additionally, we acknowledge that the number of satisfaction scores decreases over time. We ascribe this to the observational nature of the study, which reflects real-world usage and thus does not require patients to report satisfaction at any point. New patients are always being asked by their CCTs to use the app and thus those earlier in treatment are more likely to be the most represented in the first two reports, but those numbers will naturally decrease over time for a number of reasons. Certainly, those who rate the app lower may eventually decide to stop using it or decline to report satisfaction. Another subgroup is more likely to stop reporting satisfaction scores because they have completed the active treatment phase of their journey. The workflows of a number of sites require, at the cessation of active treatment, patients stop using the app because, with the transition to another service (e.g. primary care), so does the responsibility of monitoring patient needs. Thus, there is a ceiling with regard to the number of satisfaction reports patients at those sites can make. Additionally, some may become too fatigued, sick or die during treatment. Nevertheless, we think it is important to note that the inclusion of any of this information (sociodemographic, treatment, or reasons for stopping satisfaction reporting) may not impact findings given the overall high levels of satisfaction initially and longitudinally. We also chose not to report characteristics of the sites to ensure that we do not unintentionally identify them. Thus, taken together, we believe that our findings are an accurate representation of patients’ ePRO experience.

We will endeavor to conduct follow-up studies. As mentioned previously, when patients are offered the opportunity to report their satisfaction, they are also given the option to provide additional comments via a free-text box. The inclusion of this information may yield a deeper understanding of these satisfaction scores. Additionally, we look forward to collaborating with individual sites to combine satisfaction with EHR data. This will help us better clarify experiences overall, achieve better balance between systemic therapy and radiation therapy patients, and better represent those receiving more complex care such as those receiving both treatments together or sequentially.

## Conclusions

In a study of unprecedented size and scope of patients actively receiving treatment in United States, Canada, and Europe, it was found that, despite health system, practice, and ePRO deployment differences, RT and ST patients using the app to report treatment-related symptoms in real-time generally conveyed high levels of longitudinal ePRO satisfaction and that differences across the groups were negligible. It is hypothesized that these high scores imply high app utility; patients in both groups find it useful. Our study indicates that a single solution can be deployed across two widely used treatment modalities, which simplifies CCT workflow and minimizes patient burden.

## Data Availability

The datasets generated and/or analyzed during the current study are not publicly available because they are proprietary but are available from the corresponding author on reasonable request.

## Abbreviations

Abbreviation: Term
CCT: Clinical care team
CI: Confidence intervals
EMM: Estimated marginal means
EMP: Estimated marginal predictions
NB: Negative binomial mixed model
OR: Odds Ratio
RT: Radiotherapy
SD: Standard Deviation
ST: Systemic therapy
US: United States
